# Editorial: Challenging the concept of self-management support in unique and diverse populations

**DOI:** 10.3389/fresc.2022.999528

**Published:** 2022-09-05

**Authors:** Leigh Hale, Sarah Oosman, Aimee Vivienne Stewart

**Affiliations:** ^1^ Centre for Health, Activity, and Rehabilitation Research, School of Physiotherapy, University of Otago, Dunedin, New Zealand; ^2^ School of Rehabilitation Science, University of Saskatchewan, Saskatoon, SK, Canada; ^3^ Saskatchewan First Nations & Métis Health Research Network (FMRHN), Saskatoon, SK, Canada; ^4^ Department of Physiotherapy, University of the Witwatersrand, Johannesburg, South Africa

**Keywords:** self-management support, diversity, long-term conditions, cultural safety, cultural humility

**Editorial on the Research Topic** **Challenging the concept of self-management support in unique and diverse populations** by Hale L, Oosman S, and Stewart A. (2022) Front. Rehabilit. Sci. 3: 999528. doi: 10.3389/fresc.2022.999528

Self-management is arguably the healthcare buzzword of the early 21st century resulting in extensive global research. Living with a lifelong condition (health and/or disability) has an impact on the individual personally (physically, mentally, emotionally, spiritually, and economically), as well as their family, the wider society, and the economy (1, 2). Individualistic “self-management” strategies are often used as a primary health goal, but they are not always realistic, relevant, accessible, or achievable, particularly for unique and diverse populations. The impact of lifelong conditions on the health of populations, including quality of life and the associated costs, is an important determinant of public policy and public spending. Underlying the interest in and recognition of the impact of lifelong conditions are neoliberal discourses, resulting in work undertaken to reduce the burden arising from lifelong conditions (3). Thus, “defining and supporting patient self-management [has become] an important task of health services. Self-management [is now] a ‘policy relevant’ construct, clearly within the remit of the health system and [therefore] one of the daily tasks of patients and health professionals in their encounters” [(4), p. 3].

“Self-management” *per se* can be viewed as patients with lifelong conditions going it alone, applying the knowledge they receive from healthcare professionals to manage their health conditions on their own, supported by a suite of resources that help people in choosing healthy behaviours. On the other hand, “self-management support” is a person-centred approach that centres on a collaborative relationship between the person with the lifelong condition and their healthcare professionals, a relationship that enables and supports the person to manage their condition/s (5). Self-management support should be underpinned by four principles: (1) affording people dignity, compassion, and respect; (2) offering coordinated care, support, or treatment; (3) offering personalised care, support, or treatment; and (4) supporting people to recognise and develop their own strengths and abilities to enable them to live an independent and fulfilling life (6). The complexities of these fundamental principles may cause ethical dilemmas, low self-efficacy, and anxieties, resulting in possible rejection by both patients and healthcare professionals (1). These authors suggest a capabilities approach to enable true self-management support that “focuses on what it matters that people can be and do (their valued functionings) and whether individual people have the freedoms or genuine opportunities to be and do those things (whether they have the capabilities to realise those valued functionings)” [(1), p. 57). Sheridan et al. (7) in a study conducted in New Zealand and Canada entitled “How does it feel to be a problem?” concluded that little attention has been given to the “how” of self-management support, how can the components of self-management be delivered, and how can relationships between healthcare professionals and patients be intentionally nurtured to enable capabilities focused self-management support.

The multifaceted nature of these relationships creates “tension between patient autonomy, professional responsibility for the delivery of evidence-based practice”, and the funding constraints, goals, or set up of the health system [(8), p. 137]. There is an underlying assumption that people have the “agency or free will to make daily decisions that would benefit their health”, while “overlook[ing] the powerful effect of social context” and that “not everyone is in a socio-economic position to prioritise health” [(9), p. 2]. There are thus continuing inequities in access to self-management programmes or support; with certain people unable to access such programmes (due to multiple reasons, including geography, culture, ethnicity, language, physical or cognitive limitations, communication, and socio-economic status), and there are often high attrition rates from such programmes. Furthermore, to some communities that value collectivism over individualism, the concept of “self”-management is an antithesis to their values and beliefs.

In this research topic, we seek to explore the notion of “self-management support” in the context of rehabilitation in order to help unique and diverse populations live their best lives and reduce health inequities. Such populations may include people living in rural and remote regions, indigenous peoples, people of diverse genders, people with lifelong disabilities, people with cognitive challenges, or people in low socio-economic situations.

A common concept highlighted across the four papers in this series is that rehabilitation professionals need to build their own abilities and skills in order to develop relationships and pay attention to their own and others' values and biases (Wilson et al.). Rehabilitation professionals need to be open to other ways of knowing and being, beyond the entrenched Westernised biomedical approach that largely underpins rehabilitation delivery worldwide and find innovative approaches that are relevant to the communities they work with (Smith). For example, Hutchinson et al. expand in their perspective piece upon the understandings and use of leisure and leisure education to support self-management, an approach that requires education, coordinated leadership, patient navigation, and building of multi-sectoral partnerships. Wilkinson et al. provide practical suggestions for developing, delivering, sustaining, and supporting a rural or internet-delivered, community-wide, generic long-term conditions rehabilitation programme that includes co-development with community end-users, building relationships that are representative and inclusive, and being flexible in programme design and delivery. Fundamental to equity in healthcare delivery are the cultural needs of unique and diverse populations (Smith). Wilson et al., on the other hand, highlight the scarcity of research exploring professionals' perspectives on translating cultural safety concepts into practice.

This collection of papers highlights the importance of healthcare practitioners continually and critically reflecting on their practice. To enable people living with lifelong conditions (health and/or disability), rehabilitation practitioners must be able to recognise limitations that exist in their own, and within rehabilitation, practice and learn how to respond to these limitations in ways that create opportunities to learn and grow from diverse perspectives and experiences. This requires a culturally humble approach to practice. Perhaps the buzzword of healthcare in the 21st century, to enable all people with lifelong conditions (health and/or disability) to live the best life possible, should not be “self-management” but rather be reframed as “cultural humility” that supports people, their families, and larger communities' endeavours to manage their health and wellness.
